# Neuroprotective Effects of Salicin in a Gerbil Model of Transient Forebrain Ischemia by Attenuating Oxidative Stress and Activating PI3K/Akt/GSK3β Pathway

**DOI:** 10.3390/antiox10040629

**Published:** 2021-04-20

**Authors:** Joon-Ha Park, Tae-Kyeong Lee, Dae-Won Kim, Hyejin Sim, Jae-Chul Lee, Jong-Dai Kim, Ji-Hyeon Ahn, Choong-Hyun Lee, Young-Myeong Kim, Moo-Ho Won, Soo-Young Choi

**Affiliations:** 1 Department of Anatomy, College of Korean Medicine, Dongguk University, Gyeongju 38066, Gyeongbuk, Korea; jh-park@dongguk.ac.kr; 2 Department of Biomedical Science, Research Institute for Bioscience and Biotechnology, Hallym University, Chuncheon 24252, Gangwon, Korea; tk-lee@hallym.ac.kr; 3 Department of Biochemistry and Molecular Biology, Research Institute of Oral Sciences, College of Dentistry, Gangnung-Wonju National University, Gangneung 25457, Gangwon, Korea; kimdw@gwnu.ac.kr; 4 Department of Neurobiology, School of Medicine, Kangwon National University, Chuncheon 24341, Gangwon, Korea; janny20@naver.com (H.S.); anajclee@kangwon.ac.kr (J.-C.L.); jh-ahn@ysu.ac.kr (J.-H.A.); 5 Division of Food Biotechnology, School of Biotechnology, Kangwon National University, Chuncheon 24341, Gangwon, Korea; jongdai@kangwon.ac.kr; 6 Department of Physical Therapy, College of Health Science, Youngsan University, Yangsan 50510, Gyeongnam, Korea; 7 Department of Pharmacy, College of Pharmacy, Dankook University, Cheonan 31116, Chungnam, Korea; anaphy@dankook.ac.kr; 8 Department of Molecular and Cellular Biochemistry, School of Medicine, Kangwon National University, Chuncheon 24341, Gangwon, Korea; ymkim@kangwon.ac.kr

**Keywords:** salicin, transient ischemia, neuroprotection, oxidative stress, PI3K/Akt/GSK3β pathway

## Abstract

Salicin is a major natural compound of willow bark and displays diverse beneficial biological properties, such as antioxidant activity. However, little information available for the neuroprotective potential of salicin against ischemic brain injury has been reported. Thus, this study was performed to investigate the neuroprotective potential of salicin against ischemia and reperfusion (IR) injury and its mechanisms in the hippocampus using a gerbil model of 5-min transient ischemia (TI) in the forebrain, in which a massive loss (death) of pyramidal neurons cells occurred in the subfield Cornu Ammonis 1 (CA1) among the hippocampal subregions (CA1-3) at 5 days after TI. To examine neuroprotection by salicin, gerbils were pretreated with salicin alone or together with LY294002, which is a phosphatidylinositol 3-kinase (PI3K) inhibitor, once daily for 3 days before TI. Treatment with 20 mg/kg of salicin significantly protected CA1 pyramidal neurons against the ischemic injury. Treatment with 20 mg/kg of salicin significantly reduced the TI-induced increase in superoxide anion generation and lipid peroxidation in the CA1 pyramidal neurons after TI. The treatment also reinstated the TI-induced decrease in superoxide dismutases (SOD1 and SOD2), catalase, and glutathione peroxidase in the CA1 pyramidal cells after TI. Moreover, salicin treatment significantly elevated the levels of phosphorylation of Akt and glycogen synthase kinase-3β (GSK3β), which is a major downstream target of PI3K, in the ischemic CA1. Notably, the neuroprotective effect of salicin was abolished by LY294002. Taken together, these findings clearly indicate that salicin protects against ischemic brain injury by attenuating oxidative stress and activating the PI3K/Akt/GSK3β pathway.

## 1. Introduction

Transient ischemia (TI) in whole brain happens by transient interruption or reduction in cerebral blood flow and induces selective and “delayed neuronal death (DND)” in vulnerable regions, including the hippocampus, a few days later [1,2]. Previous studies have provided increasing amounts of evidence showing that the pathogenesis of DND induced by TI is associated with complex mechanisms, including glutamate-induced excitotoxicity [3,4], oxidative stress following the overgeneration of reactive oxygen species (ROS) [5,6], and glial cell-mediated inflammatory response [7,8]. However, the molecular mechanisms underlying the TI-induced DND are not yet exactly understood.

The natural compounds derived from medicinal plants have been recognized for a vital source of therapeutic agents to treat neurological disorders for recent several decades of years due to their diverse biological properties [9,10]. Salicin, a precursor to acetylsalicylic acid (aspirin), is a major active compound in willow bark that has been utilized as a traditional medicine for the treatment of diverse ailments, such as fever, mild rheumatic complaints, and pain [11,12]. Recent studies have reported that salicin possesses a broad range of pharmacological activities, including antioxidant [13], anti-inflammatory [14], anti-cancer [15], and neurite outgrowth effects [16].

There are many reports to show the neuroprotective effects of aspirin against ischemic injury in in vitro and in vivo models of cerebral ischemia [17,18,19]. However, to the best of our knowledge, the neuroprotective potential of salicin against TI-induced brain injury have not been explored in experimental models of TI in brains. Therefore, the objective of this study is to scrutinize the neuroprotective potential of salicin against ischemic injury following TI in the forebrain of the gerbil, which has been known as a good in vivo model for exploring the mechanisms of TI-induced DND and for screening the methods assessing neuroprotective agents [20,21]. For the examination of the neuroprotective mechanism by salicin, we examined the antioxidative mechanism in the neuroprotection using immunohistochemistry for superoxide anion and endogenous antioxidants. In addition, Western blot analysis was performed for the PI3K/*AKT*/GSK-3β pathway, which is known to play a crucial role in neuroprotection, cell survival by inhibiting apoptosis, and proliferation [22].

## 2. Materials and Methods

### 2.1. Experimental Animals and Protocol

Male Mongolian gerbils at 6 months of age (body weight, 70–80 g; total *n* = 145) were used in this study. They were grown and cared for in the “Experimental Animal Center” at Kangwon National University (Chuncheon, Kangwon, Korea).

Our experimental protocol including animal care and handling was approved (approval number, KW-200113-1; approval date, 18 February 2020) from the “Institutional Animal Care and Use Committee”, a committee of our University. The gerbils used in this experiment were cared for in rooms with constant temperature (about 23 °C), humidity (about 55%), and a 12 h light/dark cycle. The animal handling and care faithfully followed the guidelines included in the “Current international laws and policies” of the “NIH Guide for the Care and Use of Laboratory Animals” from The National Academies Press (8th Ed., 2011). The number of the gerbils used for this research was minimized, and the suffering caused by the procedures used in this experiment was also minimized.

### 2.2. Groups and Drug Administration

Thirty gerbils were used for the dosage to show protective effects in 10 and 20 mg/kg salicin-TI groups (*n* = 5 at each time after TI, respectively), which was treated with 10 and 20 mg/kg of salicin before TI operation and received TI operation. In addition, we examined side effects in 10 and 20 mg/kg salicin-sham groups (*n* = 5 at each time after sham TI, respectively), which was treated with 10 and 20 mg/kg of salicin before sham TI operation and received sham TI operation. The gerbils in each group were sacrificed at 0 day and 5 days after TI: at this time after TI, DND occurred in CA1. In this experiment, we observed that 20 mg/kg of salicin showed neuroprotection in CA1.

Next, 115 gerbils were used to study the mechanisms of neuroprotection by 20 mg/kg of salicin in (1) vehicle-sham group (*n* = 10) was treated with vehicle and received sham TI operation; (2) vehicle-TI group (*n* = 25) was treated with vehicle and received TI operation; (3) salicin-sham group was treated with 20 mg/kg (*n* = 10) of salicin and given sham operation; (4) salicin-TI group was treated with 20 mg/kg (*n* = 25) of salicin and received TI operation; (5) salicin/LY294002 (LY)-sham group (*n* = 15) was treated with 20 mg/kg of salicin and 1 mg/kg of LY, and received sham operation; (6) salicin/LY-TI group (*n* = 30) was treated with 20 mg/kg of salicin and 10 mg/kg of LY, and received TI operation.

### 2.3. Administration of Drugs

Salicin (Sigma-Aldrich, St. Louis, MO, USA) was dissolved in saline used as a vehicle and injected intraperitoneally once/day for 5 days before TI surgery. LY (LY294002, Sigma-Aldrich), which is an inhibitor of PI3K that is a major mode of activation of Akt (also named protein kinase B), has been widely used in studies of cerebral ischemia regarding the PI3K/Akt/GSK3β signaling pathway [23,24]. LY (10 mg/kg) was dissolved in dimethyl sulfoxide. The LY dose was chosen based on the results of a previous study showing that 10 mg/kg of LY significantly inhibited the PI3K/Akt/GSK3β pathway in the rat brain after TI [24]. The salicin/LY group was intraperitoneally administered with salicin and LY at the same time.

### 2.4. TI operation

As previously described [25], the gerbils used for TI or sham TI were anesthetized with 2.5% isoflurane (in 33% oxygen and 67% nitrous oxide). Under anesthesia, both common carotid arteries located in the carotid sheath of the neck were isolated and occluded with clips for 5 min. Ischemia (interruption of blood supply to brains) was confirmed in central arteries in both retinae with ophthalmoscope (HEINE K180) (Heine Optotechnik, Herrsching, Germany). After the conformation of perfect ischemia, the clips were removed, and blood recirculation was confirmed. Thereafter, the incised area was closed with 3–0 suture silk (Ethicon Inc., Somerville, NJ, USA). Body temperature of the ischemic gerbils was controlled at normal level (37 ± 0.5 °C) using a rectal temperature probe (TR-100) obtained from Fine Science Tools Inc (Foster City, CA, USA) during the TI operation and until the gerbils resumed their strength. The gerbils of the sham groups underwent the same TI operation without ligation of the arteries.

### 2.5. Preparation of Sections for Histopathological Observation

For histopathological examination following TI, the sections containing the hippocampus were prepared as previously described [21]. In short, the gerbils in each group (*n* = 5 at each time) were deeply anesthetized by intraperitoneal injection of 1.5 g/kg of urethane (Sigma-Aldrich) at designated times (0 day, 2 days, and 5 days after TI). Under anesthesia, the brains of the gerbils were fixed by perfusion with 4% paraformaldehyde solution (in 0.1 M phosphate-buffer (PB), pH 7.4). The fixed brains were removed and more fixed in the fixative for 4 h. Thereafter, the brain tissues were infiltrated with 30% sucrose (in 0.1 M PB) for 10 h to avoid tissue damage from freezing. Finally, the brain tissues were frontally sectioned into 30 µm of thickness in cryostat of Leica Microsystems GmbH (Wetzlar, Germany), and they were stored in phosphate-buffered saline (PBS, pH 7.4).

In this experiment, the sections with hippocampi were obtained between levels of −1.4 mm and −2.2 mm based on Bregma according to the Gerbil Brain Atlas by Radtke-Schuller (2016) [26].

### 2.6. Histochemistry with Cresyl Violet (CV)

CV histochemical staining (a method to detect Nissl’s body) was done to examine change in cells (neurons and glial cells) according to a published method [27]. In brief, the sections were stained in 0.1% CV solution (Sigma-Aldrich) for 20 min at room temperature and rinsed in distilled water. Continuously, they were decolorized in 70% ethyl alcohol for a few seconds, dehydrated in 80%, 90%, 95%, and 100% ethyl alcohol, and cleared in xylene. Finally, they were mounted with Canada balsam (Sigma-Aldrich).

### 2.7. Fluoro-Jade B (FJB) Histofluorescence Technique

Histofluorescence with FJB (a fluorescent marker of cellular degeneration or death) was carried out to examine the damage/death of neurons in the gerbil hippocampus after TI. In short, it was described in our previous study [25]. The prepared brain sections were immersed in 0.06% potassium permanganate solution and reacted in 0.0004% FJB (Histochem, Jefferson, AR, USA) solution. After that, they were rinsed in distilled water and put onto a slide warmer until they were fully dried. Finally, the slides were cleared by immersion in xylene and coverslipped with DPX (Fluka, Milwaukee, WI, USA).

The sections stained with FJB were observed using an Olympus BX53 fluorescence microscope (Deutschland GmbH, Hamburg, Germany) with blue excitation fluorescence filtered between 450 and 490 nm. Images of FJB-positive (FJB^+^) cells, which underwent degeneration and brightly fluoresced in comparison with the background [28], were captured and counted using an image analyzing system (Optimas 6.5 version) from CyberMetrics (Scottsdale, AZ, USA).

### 2.8. Dihydroethidium (DHE) Histofluorescence Technique

DHE (Sigma-Aldrich), a probe detecting superoxide anion, was used to assess in situ production of superoxide anions. DHE histofluorescence was done according to a published method [21]. Briefly, the prepared sections were equilibrated in Krebs-HEPES buffer (pH 7.4), which contained 130 mM NaCl, 5.6 mM KCl, 2 mM CaCl2, 0.24 mM MgCl2, 11 mM glucose, and 8.3 mM HEPES, for 30 min at 37 °C and immediately reacted in fresh buffer containing DHE (10 μmol/L). Finally, these sections were covered with coverglasses and stored in humidified dark chambers for 2 h at 37 °C.

As previously described [25], the intensity of DHE fluorescence was evaluated. Briefly, a digital image of DHE fluorescence was captured using an Olympus fluorescence microscope (BX53), as described above. The DHE fluorescence intensity was assessed as % using Image-pro Plus 6.0 software of Media Cybernetics Inc. (Silver Spring, MD, USA).

### 2.9. Determination of Lipid Peroxidation

ROS-induced lipid peroxidation was examined via measuring the level of 4-hydroxy-2-nonenal (4HNE, an end-product by lipid peroxidation) in accordance with previously described methods [29,30]. In brief, five gerbils per group were deeply anesthetized by intraperitoneal injection of 90 mg/kg pentobarbital sodium (JW Pharm. Co., Ltd., Seoul, Korea). Thereafter, their brains were harvested and hippocampal CA1 tissues were dissected. The obtained CA1 tissues were homogenized with a buffer containing 10 mM Hepes (pH 7.5, containing 200 mM mannitol, 70 mM sucrose, 1 mM EGTA, and 5 mM butylated hydroxytoluene) and extracted with dichloromethane. An aliquot of the lower organic phase was dried under nitrogen and rehydrated. The samples were mixed with *N*-methyl-2-phenylindole in acetonitrile and methane sulfonic acid. Thereafter, they were incubated and centrifuged. Using a Beckman DU-64 spectrophotometer (Beckman Instruments, Inc., Fullerton, CA, USA), the absorbance of the clear supernatant was measured at 586 nm wavelength. The 4HNE concentration in each sample was determined against 4HNE standard provided in the assay kit.

### 2.10. Immunohistochemical Staining

In this experiment, immunohistochemistry was performed to examine using the following primary antibodies for (1) neuronal damage or death by neuronal nuclear antigen (NeuN, a marker for neuron), (2) 4HNE, and (3) endogenous antioxidant enzymes by Cu, Zn-superoxide dismutase (SOD1), Mn-superoxide dismutase (SOD2), catalase (CAT), and glutathione peroxidase (GPX). As previously described [21], in short, the prepared brain sections were treated with 0.3% hydrogen peroxide solution and followed by 10% normal horse serum solution. Subsequently, these were incubated in each solution of primary antibody as follows: mouse anti-NeuN (dilute 1:800, Chemicon, Temecula, CA, USA), mouse anti-4HNE (dilute 1:800, Alexis Biochemicals, San Diego, CA, USA), sheep anti-SOD1 (dilute 1:800, Calbiochem, La Jolla, CA, USA), sheep anti-SOD2 (dilute 1:800, Calbiochem, La Jolla, CA, USA), rabbit anti-CAT (dilute 1:800, Abfrontier, Seoul, Korea), and rabbit anti-GPX (dilute 1:800, Chemicon, Temecula, CA, USA). Thereafter, these were reacted in biotinylated donkey anti-mouse, sheep, or rabbit immunoglobulin G (IgG) (dilute 1:250, Vector, Burlingame, CA, USA) and avidin–biotin complex (dilute 1:200, Vector, Burlingame, CA, USA). Finally, these immunoreacted sections were visualized by soaking them in 3,3′-diaminobenzidine tetrahydrochloride (Sigma-Aldrich) solution.

In this experiment, to evaluate the specificity of immunostaining, a negative control test was performed with preimmune serum in place of each primary antibody. The test sections showed no immunoreaction in the brain sections (data not shown).

To examine survival of neurons, NeuN^+^ cells were counted according to a previously published method [25]. Briefly, the images of NeuN^+^ cells were taken using an Olympus BX53 microscope. The count of NeuN^+^ cells was obtained by averaging total numbers using an image analyzing system, as described above.

To examine oxidative stress and antioxidative effects, changes in the immunoreactivity of 4HNE, SOD1, SOD2, CAT, and GPX were evaluated according to our previous method [25]. In brief, digital images of each immunoreactive structure were obtained, as described above. Changes in each immunoreactivity were assessed on the basis of relative immunoreactivity (RI) of 4HNE, SOD1, SOD2, CAT, and GPX. Namely, the RI was calibrated as % using Adobe Photoshop version 8.0 (San Jose, CA, USA) and NIH Image 1.59 software (Bethesda, MD, USA).

### 2.11. Western Blot Technique

To analyze proteins involved in (1) SOD1, SOD2, CAT, and GPX, and (2) the PI3K/Akt/GSK3β pathway in CA1 after TI, the gerbils (*n* = 5 at each time in each group) were sacrificed at 0 day, 2 days, and 5 days after TI. As previously described [21], the gerbils were deeply anesthetized, as described in the section of histopathology. The brains were removed as rapidly as possible and were transversely cut into about 1 mm of thicknesses. Immediately, under an enlarger, CA1 was dissected with a surgical blade. The obtained CA1 tissues were homogenized in 50 mM PBS (pH 7.4), which contained 10 mM ethylendiamine tetraacetic acid (pH 8.0), 0.1 mM ethylene glycol bis (2-aminoethyl ether)-N,N,N′,N′ tetraacetic acid (pH 8.0), 0.2% Nonidet P-40, 15 mM sodium pyrophosphate, 50 mM NaF, 2 mM sodium orthovanadate, 100 mM β-glycerophosphate, 150 mM NaCl, 1 mM phenylmethylsulfonyl fluoride, and 1 mM dithiothreitol. The homogenates were separated by centrifugation, and each protein level was established in each supernatant using a Micro BCA protein assay kit from Pierce Chemical (Rockford, IL, USA). Aliquots containing total protein were boiled in loading buffer, which contained 150 mM Tris (pH 6.8), 0.3% bromophenol blue, 6% sodium dodecyl sulfate (SDS), 3 mM DTT (full name), and 30% glycerol, and then loaded onto 12.5% polyacrylamide gel. Subsequently, the aliquot was subjected to electrophoresis, and the gel was transferred to nitrocellulose transfer membranes of Pall Corp (Pittsburgh, PA, USA). Continuously, it was blocked with 5% bovine serum albumin, and the membrane was incubated with each primary antibody: goat anti-4-HNE (dilute 1:5000, Abcam, Cambridge, UK), rabbit anti-SOD2 (dilute 1:2000, Abcam, Cambridge, UK), rabbit anti-SOD2 (dilute 1:2000, Abcam, Cambridge, UK), rabbit anti-CAT (dilute 1:2000, Merck KGaA, Darmstadt, Germany), rabbit anti-GPX (dilute 1:2000, Abcam, Cambridge, UK) rabbit anti-Akt (dilute 2:000, Cell Signaling Technology, Danvers, MA, USA), rabbit-phospho-Akt (Ser473) (dilute 1:2000, Cell Signaling Technology), rabbit anti-GSK3β (dilute 1:1000, Cell Signaling Technology), rabbit-phospho-GSK3β (Ser9) (dilute 1:1000, Cell Signaling Technology), and rabbit anti-β-actin (dilute 1:2,000, Sigma-Aldrich) for 10 h at 4 °C. Finally, it was reacted to peroxidase conjugated goat anti-rabbit IgG of Santa Cruz Biotechnology (dilute 1:4000, Heidelberg, Germany) and an enhanced chemiluminescence kit of GE Healthcare Life Sciences (Chalfont, UK).

The assessment of Western blotting was done according to a published method [31]. In brief, the Western band was scanned using ChemiDoc Imaging System of Bio-Rad Laboratories Inc (Hercules, CA, USA), and the quantification of the analysis was performed using Scion Image software of Scion Corp (Frederick, MD, USA). The phosphorylation level of the targeted protein was evaluated by comparing with corresponding total protein. β-actin was used as an internal control.

### 2.12. Statistical Analysis

Data obtained here are presented as the means ± standard error of the mean (SEM). All statistical analyses were done with the aid of GraphPad Prism (version 5.0) of GraphPad Software (La Jolla, CA, USA). To test normal distributions, we used the Kolmogorov and Smirnov test and used Bartlett test for testing identical SEMs. Additionally, all the presented data passed the normality test. The differences of the means among all groups were statistically analyzed by two-way analysis of variance (ANOVA) with a post hoc Bonferroni’s multiple comparison test to elucidate TI-mediated differences among all of the groups. For statistical significance, *p* < 0.05 was used.

## 3. Results

### 3.1. Salicin Saved Hippocampal Neurons from TI Injury

#### 3.1.1. CV^+^ Cells

**Figure 1:**
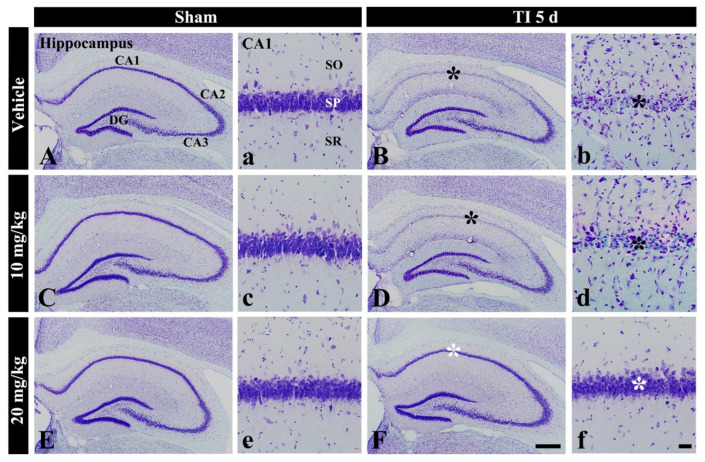
CV staining in the hippocampus of the vehicle-sham (**A**,**a**), vehicle-TI (**B**,**b**), 10 mg/kg salicin-sham (**C**,**c**), 10 mg/kg salicin-TI (**D**,**d**), 20 mg/kg salicin-sham (**E**,**e**), and 20 mg/kg salicin-TI (**F**,**f**) groups at 5 days post-TI. In the vehicle-TI and 10 mg/kg salicin-TI groups, CV stainability in the stratum pyramidale (SP, black asterisks) of CA1 is very pale. However, in the 20 mg/kg salicin-TI group, strong CV stainability is observed in the SP (white asterisks) (*n* = 5/group; the vehicle-sham group was regarded as control group). DG, dentate gyrus. SO, stratum oriens; SR, stratum radiatum. Scale bar = 400 μm (**A**–**F**) and 50 μm (**a**–**f**).

In the vehicle-sham group, cells stained with CV were well defined in all subregions (CA1-3) of the hippocampus (Figure 1A(a)). In the 10 and 20 mg/kg salicin-sham groups, no significant difference in CV stainability was found compared with that in the vehicle-sham group (Figure 1C(c),E(e)).

In the vehicle-TI group, CV stainability was distinctly decreased in cells of the stratum pyramidale (called pyramidal neurons or cells) in CA1 at 5 days post-TI (Figure 1B(b)). This result means that pyramidal cells located in CA1 were damaged by TI. In the 10 mg/kg salicin-TI group, CV stainability in CA1 pyramidal cells was not different from that in the vehicle-TI group (Figure 1D(d)). However, in the 20 mg/kg salicin-TI group, CV stainability in CA1 pyramidal neurons was similar to that in the vehicle-sham group (Figure 1F(f)). Based on these findings, 20 mg/kg of salicin was used to investigate neuroprotection and its related mechanisms, as below.

#### 3.1.2. NeuN^+^ and F-J B^+^ Cells

**Figure 2:**
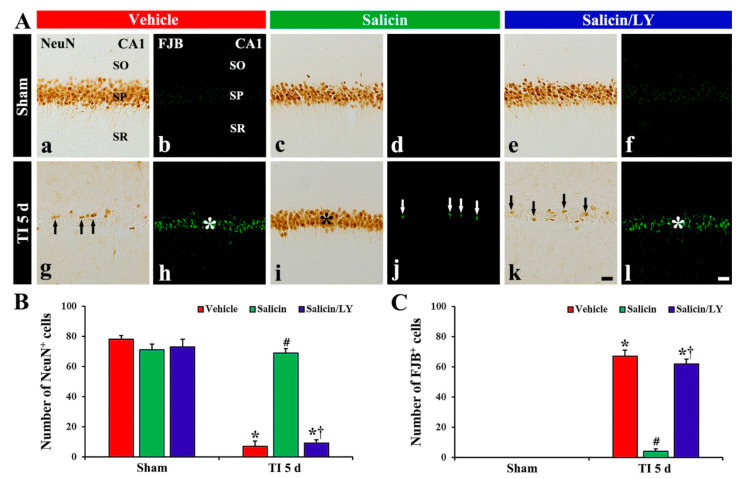
(**A**) NeuN immunohistochemistry (**a**,**c**,**e**,**g**,**i**,**k**) and FJB histofluorescence (**b**,**d**,**f**,**h**,**j**,**l**) in CA1 of the vehicle-sham (**a**,**b**), vehicle-TI (**g**,**h**), salicin-sham (**c**,**d**), salicin-TI (**i**,**j**), salicin/LY-sham (**e**,**f**), and salicin/LY-TI (**k**,**l**) groups at 5 days post-TI. A few NeuN^+^ pyramidal cells (black arrows) and many FJB^+^ pyramidal cells (white asterisk) are detected in the vehicle-TI group. In the salicin-TI group, however, abundant NeuN^+^ cells (black asterisk) and a few F–J B^+^ cells (white arrows) are observed in the SP. On the contrary, in the salicin/LY-TI group, the distribution pattern and number of NeuN^+^ and FJB^+^ cells (black arrows and white asterisk) in the SP are similar to those in the vehicle-TI group. Scale bar = 50 μm. (**B**,**C**) The mean numbers of NeuN^+^ pyramidal neurons (**B**) and FJB^+^ pyramidal neurons (**C**) in CA1. The bars indicate the means ± SEM (*n* = 5/group, * *p* < 0.05 vs. each sham group, **^#^** *p* < 0.05 vs. vehicle-TI group, **^†^** *p* < 0.05 vs. salicin-TI group; the vehicle-sham group was regarded as control group).

In the vehicle-sham group, clear NeuN immunoreactivity was shown in the CA1 pyramidal cells (Figure 2A(a)), and no FJB histofluorescence was detected in any cells (Figure 2A(b)). In the salicin-sham group, the distribution pattern and number of NeuN^+^ pyramidal cells were similar to those shown in the vehicle-sham group (Figure 2A(c),B). In addition, FJB histofluorescence was also not observed in CA1 (Figure 2A(d)).

In the vehicle-TI group, a significant decrease (7 cells/250 μm^2^) in numbers of NeuN^+^ pyramidal neurons was shown at 5 days post-TI (Figure 2A(g),B), and, at this time, many FJB^+^ pyramidal cells (67 cells/250 μm^2^) were detected (Figure 2A(h),C). This finding means that most of the CA1 pyramidal neurons died at 5 days post-TI. In the salicin-TI group, the distribution pattern and number of NeuN^+^ pyramidal cells were similar to that found in the vehicle-sham group (Figure 2A(i),B), and a few FJB^+^ pyramidal cells (4 cells/250 μm^2^) were detected compared with that in the vehicle-TI group (Figure 2A(j),C). This finding means that therapeutic treatment of salicin saves the CA1 pyramidal neurons from TI injury.

### 3.2. Neuroprotective Effect of Salicin Was Abolished by LY

In the salicin/LY-sham group, the distribution and numbers of NeuN^+^ CA1 pyramidal neurons were not different from those shown in the vehicle-sham group (Figure 2A(c),A(e),B), and FJB histofluorescence was not shown in CA1 (Figure 2A(f)). This finding means that LY did not affect neurons in the sham group.

In the salicin/LY-TI group, NeuN^+^ CA1 pyramidal cells (9 cells/250 μm^2^) were significantly decreased and FJB^+^ CA1 pyramidal cells (62 cells/250 μm^2^) were significantly increased when compared with those in the salicin-sham group (*p* < 0.05) (Figure 2A(k),A(l),B,C). This finding means that LY, as an inhibitor of PI3K, inhibited the neuroprotective effect of salicin.

### 3.3. Salicin Attenuated TI-Induced Oxidative Stress, and Its Effect Was Abolished by LY

#### 3.3.1. HNE Level

**Figure 3:**
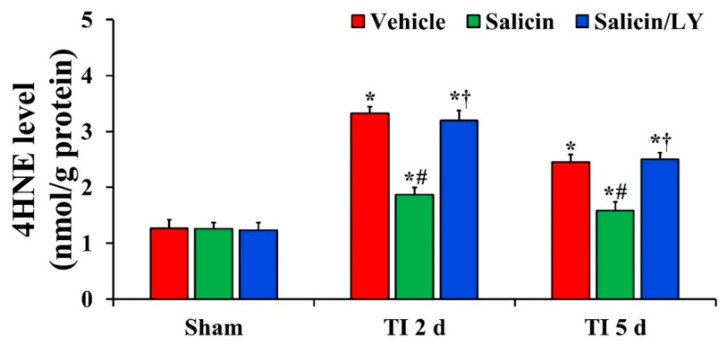
Level of 4HNE in the vehicle-treated, salicin-treated, and salicin/LY-treated groups at sham, 2 days, and 5 days after TI. In the salicin-TI group, 4HNE level is significantly reduced compared with that at the corresponding time of the vehicle-TI group. However, in the salicin/LY-TI group, 4HNE level is similar to that in the vehicle-TI group. The bars indicate the means ± SEM (*n* = 5 at each time, * *p* < 0.05 vs. each sham group, **^#^** *p* < 0.05 vs. vehicle-TI group, ^†^ *p* < 0.05 vs. salicin-TI group; the vehicle-sham group was regarded as control group).

In all sham groups, there was no significant difference in 4HNE level in CA1 (Figure 3). 4HNE level in the vehicle-TI group was significantly increased at 2 days and 5 days post-TI (Figure 3). In the salicin-TI group, 4HNE level was significantly decreased at 2 days and 5 days post-TI compared with that at the corresponding time point of the vehicle-TI group (Figure 3). On the other hand, in the salicin/LY-TI group, the pattern of change in 4HNE level was similar to that in the vehicle-TI group (Figure 3).

#### 3.3.2. DHE Fluorescence

**Figure 4:**
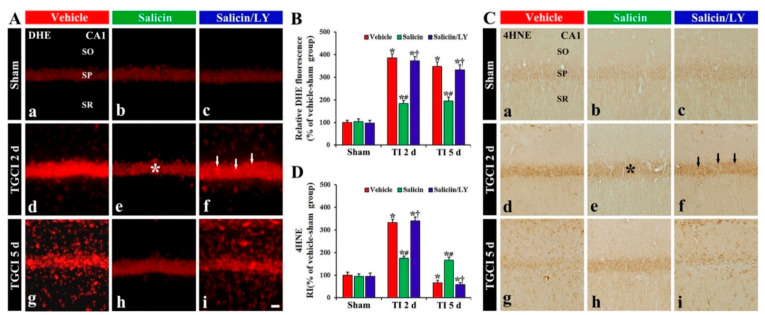
(**A**,**C**) DHE fluorescence (**A**) and 4HNE immunohistochemistry (**C**) in CA1 of the vehicle-treated (left columns), salicin-treated (middle columns), and salicin/LY-treated (right columns) groups at sham (**Aa**–**Ac**,**Ca**–**Cc**), 2 days (**Ad**–**Af**,**Cd**–**Cf**), and 5 days (**Ag**–**Ai**,**Cg**–**Ci**) after TI. In the salicin-TI group, DHE fluorescence and 4HNE immunoreactivity in pyramidal cells (asterisks) are significantly lower than that in the vehicle-TI group. However, in the salicin/LY-TI group, DHE fluorescence (white arrows) and 4HNE immunoreactivity (black arrows) are significantly higher than that in the salicin-TI group. Scale bar = 50 μm. (**B**,**D**) RI of DHE fluorescence (**B**) and 4HNE (**D**) in pyramidal cells. The bars indicate the means ± SEM (*n* = 5 at each time, * *p* < 0.05 vs. each sham group, **^#^** *p* < 0.05 vs. vehicle-TI group, **^†^** *p* < 0.05 vs. salicin-TI group; the vehicle-sham group was regarded as control group).

Weak DHE (a probe detecting superoxide anion) fluorescence was found in the CA1 pyramidal cells of the vehicle-sham group (Figure 4A(a)). In the salicin-sham group, DHE fluorescence in the pyramidal neurons was not different from that found in the vehicle-sham group (Figure 4A(b),B).

DHE fluorescence in the pyramidal neurons, in the vehicle-TI group, was significantly increased (about 385% and 349%, respectively, of the vehicle-sham group) at 2 days and 5 days post-TI (*p* < 0.05) (Figure 4A(d),A(g),B). In particular, at 5 days post-TI, strong DHE fluorescence was expressed in cells located in the stratum oriens (SO) and radiatum (SR) (Figure 4A(g)). However, in the salicin-TI group, DHE fluorescence in the pyramidal neurons at 2 days post-TI was significantly low (about 48% of vehicle-TI group) compared with that shown in the vehicle-TI group (*p* < 0.05) (Figure 4A(e),B), and the fluorescence at 5 days post-TI was not altered (Figure 4A(h),B). Interestingly, at 5 days post-TI, the cells located in the SO and SR did not show DHE fluorescence (Figure 4A(h)).

In the salicin/LY-TI group, the change of DHE fluorescence in CA1 after TI was similar to that in the vehicle-TI group (Figure 4A(f),A(i),B), showing that, in the salicin/LY-sham group, DHE fluorescence was not different from that found in the vehicle-sham group (Figure 4A(c),B).

#### 3.3.3. HNE Immunoreactivity

In the vehicle-sham group, weak 4HNE (a marker for lipid peroxidation) immunoreactivity was shown in the pyramidal cells (Figure 4C(a)). In the salicin-sham group, 4HNE immunoreactivity in the pyramidal neurons did not differ from that shown in the vehicle-sham group (Figure 4C(b),D).

In the vehicle-TI group, 4HNE immunoreactivity was significantly increased in the pyramidal cells at 2 days post-TI by about 234% compared with the vehicle-sham group (*p* < 0.05) (Figure 4C(d),D). At 5 days post-TI, 4HNE immunoreactivity in the pyramidal neurons was very low due to the death of the pyramidal neurons by TI (Figure 4C(g),D). However, in the salicin-TI group, 4HNE immunoreactivity in the pyramidal cells, at 2 days post-TI, was significantly low (about 53%) compared with that in the vehicle-TI group (*p* < 0.05) (Figure 4C(e),D). At 5 days post-TI, the decreased 4HNE immunoreactivity was not changed at 5 days post-TI (Figure 4C(h),D).

In the salicin/LY-TI group, the change pattern of 4HNE immunoreactivity in the pyramidal cells after TI was similar to that found in the vehicle-TI group (Figure 4C(f),C(i),D), showing that, in the salicin/LY-sham group, 4HNE immunoreactivity was also similar to that shown in the vehicle-sham group (Figure 4C(c),D).

### 3.4. Salicin Prevented TI-Induced Decrease of Antioxidant Enzyme Expression, and Its Effect Was Abolished by LY

#### 3.4.1. SOD1 and SOD2 Immunoreactivity

**Figure 5:**
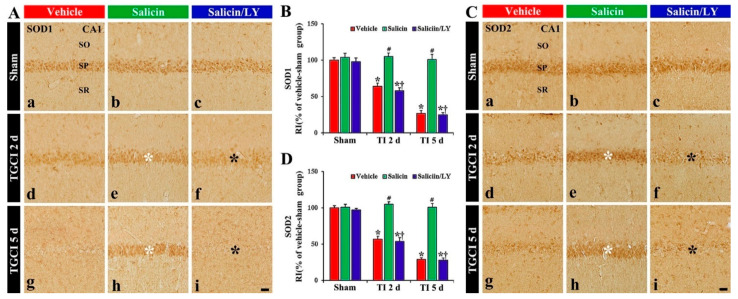
(**A**,**C**) SOD1 (**A**) and SOD2 (**C**) immunohistochemistry in CA1 of the vehicle-treated (left columns), salicin-treated (middle columns), and salicin/LY-treated (right columns) groups at sham (**Aa**–**Ac**,**Ca**–**Cc**), 2 days (**Ad**–**Af**,**Cd**–**Cf**), and 5 days (**Ag**–**Ai**,**Cg**–**Ci**) after TI. In the salicin-TI group, SOD1 and SOD2 immunoreactivity are not altered in CA1 pyramidal cells (white asterisks). However, in the salicin/LY-TI group, the two immunoreactivities (black asterisks) are significantly decreased similar to those in the vehicle-TI group. Scale bar = 50 μm. (**B**,**D**) RI of SOD1 (**B**) and SOD2 (**D**) in the pyramidal cells. The bars indicate the means ± SEM (*n* = 5 at each time, * *p*< 0.05 vs. each sham group, **^#^** *p* < 0.05 vs. vehicle-TI group, **^†^** *p* < 0.05 vs. salicin-TI group; the vehicle-sham group was regarded as control group).

In the vehicle-sham group, SOD1 and SOD2 immunoreactivity were easily shown in the pyramidal cells (Figure 5A(a),C(a)). In the salicin-sham group, SOD1, and SOD2 immunoreactivity the pyramidal cells were not different from those shown in the vehicle-sham group (Figure 5A(b),B,C(b),D).

In the vehicle-TI group, SOD1 and SOD2 immunoreactivities in the pyramidal cells were significantly reduced at 2 days post-TI by about 36% and 43%, respectively, compared with those of the vehicle-sham group (*p* < 0.05) (Figure 5A(d),B,C(d),D). At 5 days post-TI, the two immunoreactivities in the pyramidal neurons were hardly observed because of the TI-induced death of the pyramidal cells (Figure 5A(g),B,C(g),D). However, in the salicin-TI group, SOD1 and SOD2 immunoreactivity in the pyramidal cells were not reduced, showing that each RI was about 164% and 184%, respectively, at 2 days post-TI compared with that in the vehicle-TI group (*p* < 0.05) (Figure 5A(e),B,C(e),D). At 5 days in this group, SOD1 and SOD2 immunoreactivity were maintained (Figure 5A(h),B,C(h),D).

In contrast, in the salicin/LY-TI group, SOD1 and SOD2 immunoreactivity in the pyramidal cells at 2 days and 5 days post-TI were similar to those in the vehicle-TI group (Figure 5A(f),A(i),B,C(f),C(i),D), showing that, in the salicin/LY-sham group, SOD1 and SOD2 immunoreactivity were similar to those in the vehicle-sham group (Figure 5A(c),B,B(c),D).

#### 3.4.2. CAT and GPX Immunoreactivity

**Figure 6:**
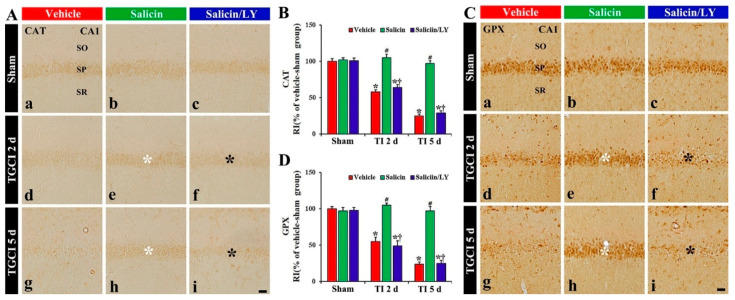
(**A**,**C**) CAT (**A**) and GPX (**B**) immunohistochemistry in CA1 of the vehicle-treated (left columns), salicin-treated (middle columns), and salicin/LY-treated (right columns) groups at sham (**Aa**–**Ac**,**Ca**–**Cc**), 2 days (**Ad**–**Af**,**Cd**–**Cf**), and 5 days (**Ag**–**Ai**,**Cg**–**Ci**) after TI. In the salicin-TI group, CAT and GPX immunoreactivity are not decreased in the pyramidal cells (white asterisks) after TI. However, the two immunoreactivities (black asterisks) in the salicin/LY-TI group are significantly reduced similar to those in the vehicle-TI group. Scale bar = 50 μm. (**B**,**D**) RI of CAT (**B**) and GPX (**D**) in the pyramidal cells. The bars indicate the means ± SEM (*n* = 5 at each time, * *p* < 0.05 vs. each sham group, **^#^** *p* < 0.05 vs. vehicle-TI group, ^†^ *p* < 0.05 vs. salicin-TI group; the vehicle-sham group was regarded as control group).

In the vehicle-sham and salicin-sham groups, CAT and GPX immunoreactivity were principally shown in the pyramidal cells, and each immunoreactivity was similar in the two sham groups (Figure 6A(a),A(b),B,C(a),C(b),D).

In the vehicle-TI group, CAT and GPX immunoreactivity in the pyramidal cells at 2 days post-TI was not altered when compared with that shown in the vehicle-sham group, showing that each RI was about 158% and 155%, respectively, of that in the vehicle-sham group (*p* < 0.05) (Figure 6A(d),B,C(d),D), and at 5 days after TI, each immunoreactivity in the pyramidal cells was hardly observed due to the death of the pyramidal cells (Figure 6A(g),B,C(g),D). However, in the salicin-TI group, CAT and GPX immunoreactivity in the pyramidal cells at 2 days post-TI were not decreased, showing that each RI was about 181% and 187%, respectively, of that in the vehicle-TI group (*p* < 0.05) (Figure 6A(e),B,C(e),D). At 5 days after TI, in this group, each immunoreactivity was sustained (Figure 6A(g),B,C(g),D).

On the other hand, in the salicin/LY-TI group, the change of CAT and GPX immunoreactivity in the CA1 pyramidal cells following TI did not differ from that in the vehicle-TI group (Figure 6A(f),A(i),B,C(f),C(i),D).

#### 3.4.3. Protein Levels of SOD1, SOD2, CAT, and GPX

**Figure 7:**
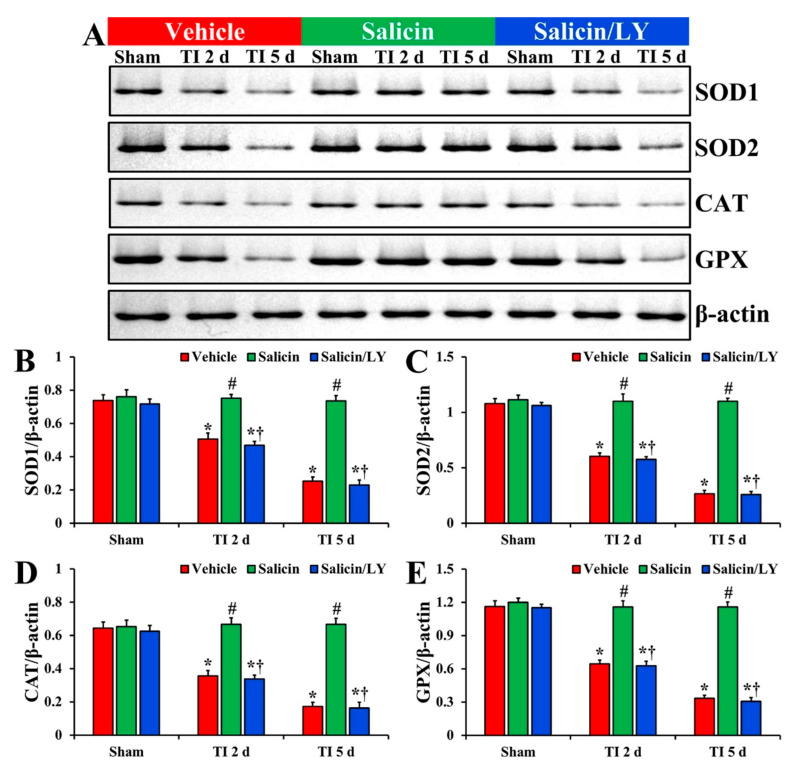
(**A**) Western blot images of SOD1, SOD2, CAT, and GPX in CA1 of the vehicle-treated, salicin-treated, and salicin/LY-treated groups at sham, 2 days, and 5 days after TI. In the salicin-TI group, each SOD1, SOD2, CAT, and GPX level is significantly higher than that in the vehicle-TI group. While in the salicin/LY-TI group, each SOD1, SOD2, CAT, and GPX level is similar to that in the vehicle-TI group. (**B**–**E**) Quantitative analyses of SOD1 (**B**), SOD2 (**C**), CAT (**D**), and GPX (**E**) level by normalization to the level of β-actin, respectively. The bars indicate the means ± SEM (*n* = 5 at each time, * *p* < 0.05 vs. each sham group, **^#^** *p* < 0.05 vs. vehicle-TI group, **^†^** *p* < 0.05 vs. salicin-TI group; the vehicle-sham group was regarded as control group).

In the vehicle-sham group, the protein levels of SOD1, SOD2, CAT, and GPX were fundamentally detected in CA1 (Figure 7A–E). In the salicin-sham group, the protein levels of the four endogenous enzymes were similar to those revealed in the vehicle-sham group (Figure 7A–E).

In the vehicle-TI group, each SOD1, SOD2, CAT, and GPX protein level was significantly lowered at 2 days post-TI (68.6%, 55.9%, 55.2%, and 55.5%, respectively vs. the vehicle-sham group) compared with each level shown in the vehicle-sham group (*p* < 0.05) (Figure 7A–E). At 5 days post-TI, each SOD1, SOD2, CAT, and GPX level was more significantly reduced (34.3%, 24.7%, 26.9%, and 28.9%, respectively vs. the vehicle-sham group) compared with each of the vehicle-sham groups (*p* < 0.05) (Figure 7A–E).

On the other hand, in the salicin-TI group, each SOD1, SOD2, CAT, and GPX level at 2 days post-TI was not significantly different (148.3%, 182.2%, 187.4%, and 179.9%, respectively vs. the corresponding time point of the vehicle-TI group) from each shown in the vehicle-sham groups (*p* < 0.05) (Figure 7A–E). Each protein level was maintained (289.7%, 412.9%, 385.3%, and 345.0%, respectively vs. the corresponding time point of the vehicle-TI group) until 5 days post-TI (*p* < 0.05) (Figure 7A–E).

In the salicin/LY-TI group, change pattern in each protein level was similar to that shown in the vehicle-TI group at 2 days and 5 days post-TI (*p* < 0.05) (Figure 7A–E).

### 3.5. Salicin Increased Phosphorylation of Akt and GSK3β after TI, and Its Effect Was Counteracted by LY

**Figure 8:**
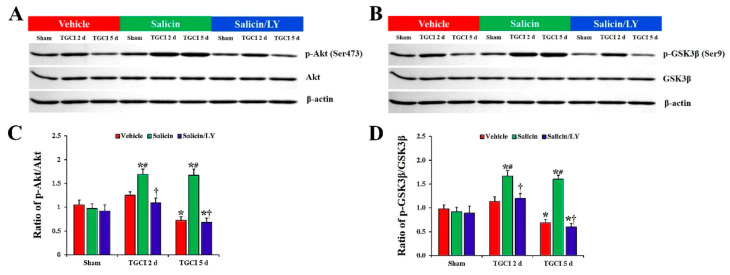
(**A**,**B**) Western blot images of p-Akt, Akt, p-GSK3β, and GSK3β in CA1 of the vehicle-treated, salicin-treated, and salicin/LY-treated groups at sham, 2 days, and 5 days after TI. In the salicin-TI group, the ratios of p-Akt/Akt and p-GSK3β/GSK3β are significantly higher than those in the vehicle-TI group. Whereas, in the salicin/LY-TI group, the ratios of p-Akt/Akt and p-GSK3β/GSK3β are similar to those in the vehicle-TI group. (**C**,**D**) Quantitative analysis of the levels of p-Akt and p-GSK3β by normalization to the levels of Akt and GSK3β, respectively. The bars indicate the means ± SEM (*n* = 5 at each time, * *p* < 0.05 vs. each sham group, **^#^** *p* < 0.05 vs. vehicle-TI group, **^†^** *p* < 0.05 vs. salicin-TI group; the vehicle-sham group was regarded as control group).

As depicted in Figure 8A–D, there were no differences in the total protein levels of Akt and GSK3β in CA1 between all experimental groups. In addition, no significant differences in protein levels of phosphor-Akt (Ser473) and phosphor-GSK3β (Ser9) in CA1 between all sham groups were found.

However, the protein levels of phospho-Akt (Ser473) and phospho-GSKβ3 (Ser9) were altered in CA1 after TI. In the vehicle-TI group, protein levels of phosphor-Akt and phosphor-GSK3β were slightly increased at 2 days post-TI compared with those in the vehicle-sham group, and each level was significantly decreased (0.7-fold and 0.6-fold, respectively) at 5 days post-TI compared with those in the vehicle-sham group (*p* < 0.05). However, in the salicin-TI group, the protein levels of phosphor-Akt and phosphor-GSK3β at 2 days post-TI were significantly increased (1.4-fold and 1.5-fold, respectively) compared to those in the vehicle-TI group (*p* < 0.05), and each protein level was maintained until 5 days post-TI.

In the salicin/LY-TI group, on the other hand, the changes of the protein levels of phosphor-Akt and phosphor-GSK3β after TI were very similar to those in the vehicle-TI group.

## 4. Discussion

Many studies have demonstrated the neuroprotective potential of medicinal plant-derived natural compounds in experimental animal models of cerebral ischemia [32,33,34]. Here, we, for the first time, examined the neuroprotective potential of salicin in a gerbil model of TI in the forebrain, which results in a massive loss (death) of pyramidal cells in the hippocampal CA1 from 4–5 days after TI [2,35]. In the current study, the results of CV histochemistry, NeuN immunohistochemistry, and FJB histofluorescence showed that pretreatment with 20 mg/kg of salicin effectively protected CA1 pyramidal cells from TI-induced brain injury. This is the first study to show the neuroprotective potential of salicin against ischemic brain injury, and this result indicates that salicin can be developed as a preventive medicine against brain ischemic insults.

Accumulating evidence has proven that oxidative stress plays a critical role in the pathogenesis of cerebral ischemia [5,36]. Oxidative stress is caused by excess production of ROS during cerebral ischemia, which can damage important cellular biomolecules, such as nucleic acids, proteins, and lipids, eventually leading to neuronal death [37,38]. Therefore, the reduction in oxidative stress is considered as a target for neuroprotection against ischemic brain injury [39]. In this study, we found that TI-induced increases in DHE (a detector of superoxide anion) fluorescence and 4HNE (a marker of lipid peroxidation) immunoreactivity in the CA1 pyramidal cells were significantly decreased by pretreatment with 20 mg/kg of salicin. For the prevention of the oxidative stress following TI, it was reported that, in a gerbil model of transient forebrain ischemia, pretreatment with chlorogenic acid (an ester of caffeic acid and quinic acid) naturally found in green coffee extract and tea significantly inhibited DHE fluorescence 4HNE immunoreactivity in ischemic hippocampal neurons [40].

There is a large body of evidence demonstrating that endogenous antioxidant enzymes, such as SODs, CAT, and GPX, play an important role in attenuating ischemic brain injury. For instance, brain injury induced by transient focal cerebral ischemia was significantly alleviated in transgenic mouse models overexpressing SOD1, SOD2, CAT, or GPX [41,42,43,44]. In addition, it was reported that pretreatments with some herbal extracts or their pure components in gerbil and rat models of TI restored TI-induced decreases in SOD1, SOD2, CAT, and GPX expressions, showing that they displayed neuroprotection against the ischemic injury [40,45,46]. For example, pretreated chlorogenic acid increased the expression levels of SOD2 in ischemic hippocampus and protected hippocampal neurons from ischemic injury induced by TI in gerbils [40]. In our current study, pretreatment with 20 mg/kg of salicin maintained the expressions of SOD1, SOD2, CAT, and GPX in the CA1 pyramidal cells after TI, although their expressions were dramatically reduced in the vehicle-TI group. For the neuroprotection by willow bark extract (WBE), it was reported that WBE prevented oxidative-stress-induced cytotoxicity of human umbilical vein endothelial cells (HUVECs), showing that WBE dose-dependently increased intracellular glutathione in the HUVECs [47]. Taken together, we suggest that antioxidative effects of salicin may contribute to the protection or attenuation of TI-induced CA1 pyramidal cell death.

It is well accepted that the PI3K/Akt/GSK3β pathway is involved in neuroprotection against ischemic brain injury [24,48,49]. Akt is phosphorylated at Ser473 residues by PI3K, which means the activation of Akt [50]. Activated Akt phosphorylates certain downstream proteins, such as GSK3β, and the phosphorylation of GSK3β sustains this protein as an inactive state [51]. The inactivation of GSK3β promotes neuronal survival after cerebral ischemia [52,53]. Endo et al. (2006) showed that the phosphorylation of Akt (Ser473) and GSK3β (Ser9) was temporally increased in the hippocampal CA1 of the rat after TI and suggested that increase in phosphor-Akt and phosphor-GSK3β might mediate survival of CA1 pyramidal cells after TI [23]. They also showed that the administration of a PI3K inhibitor reduced the phosphorylation of Akt and GSK3β and facilitated the delayed death of CA1 pyramidal cells following TI [23]. Furthermore, some studies demonstrated that pharmacological increase in the phosphorylation of Akt and GSK3β protected against brain injuries induced by transient focal and global cerebral ischemia, and its neuroprotective effect was counteracted by administration of a PI3K inhibitor [24,54,55]. Based on these relevant studies, we hypothesized that activation of the PI3K/Akt/GSK3β pathway following salicin pretreatment could play a key role in neuroprotection against ischemic injury. In our current study, we found that the levels of phosphor-Akt and phosphor-GSK3β in CA1 of the salicin-TI gerbils were significantly higher than those in the vehicle-TI group. More importantly, we found that the inhibition of the PI3K/Akt/GSK3β pathway by administration of PI3K inhibitor LY to the salicin-TI gerbils resulted in the abrogation of the neuroprotective effects observed in the salicin-TI group. Thus, our result indicates that the protective effects of pretreated salicin against TI may be mediated by activation of the PI3K/Akt/GSK3β pathway.

## 5. Conclusions

In this study, we provided evidence that pretreated salicin effectively protected pyramidal cells located in the hippocampal CA1 from TI induced by 5 min occlusion of both common carotid arteries. In the surviving CA1 pyramidal cells by salicin, oxidative stress (superoxide anion generation and lipid peroxidation) was significantly attenuated after TI. In addition, salicin pretreatment reinstated TI-induced decrease in SOD1, SOD2, CAT, and GPX. Moreover, the levels of p-Akt and p-GSK3β in CA1 of the salicin-TI gerbils were significantly high compared with those in the vehicle-TI group. Our current findings suggest that salicin can be a valuable neuroprotective candidate against ischemic injury due to its antioxidant efficacy.

## Data Availability

The data presented in this study are available on request from the corresponding author.

## Abbreviations

CA1, cornu ammonis 1; CAT, catalase; CV, cresyl violet; DHE, dihydroethidium; FJB, fluoro-jade B; GPX, glutathione peroxidase; GSK3β, glycogen synthase kinase-3β; 4HNE, 4-hydroxy-2-nonenal; LY, LY294002; NeuN, neuronal nuclear antigen; OD, optical immunoreactivity; PI3K, phosphoinositide 3-kinase; RI, relative immunoreactivity; ROS, reactive oxygen species; SOD1, Cu, Zn-superoxide dismutase; SOD2, Mn-superoxide dismutase; TI, transient ischemia
